# Population Genetic Data for 23 STR Loci of the Garifuna Ethnic Group in Honduras

**DOI:** 10.3390/genes17040402

**Published:** 2026-03-31

**Authors:** Antonieta Zuniga, Yolly Molina, Karen Amaya, Zintia Moya, Patricia Soriano, Digna Pineda, Yessica Pinto, Oscar Garcia, Isaac Zablah

**Affiliations:** 1 Dirección de Medicina Forense, Ministerio Público, Calle la Salud, Tegucigalpa 11101, Hondurasyolly.molina@unah.edu.hn (Y.M.);; 2 Center for Biomedical Imaging Diagnostics Research and Rehabilitation, National Autonomous University of Honduras, Blvd. Suyapa, Tegucigalpa 11101, Honduras; 3 Basque Country Forensic Genetics Laboratory, Larrauri Mendotxe 18, 48950 Erandia, Spain; ogarcia@seg.euskadi.eus; 4 Faculty of Medical Sciences, National Autonomous University of Honduras, Calle la Salud, Tegucigalpa 11101, Honduras

**Keywords:** short tandem repeats (STRs), population study, Garifuna, Honduras, forensic genetics, PowerPlex Fusion 6C, indigenous population, genetic markers

## Abstract

**Background:** The Garifunas are a distinctive Afro-indigenous community of Honduras, originating from the historical admixture of Island Carib, Arawak, and West African peoples in the seventeenth-century Caribbean. With an estimated 43,111 individuals residing primarily along the northern Atlantic coast. Their dual ancestral composition yields a genetic profile that differs meaningfully from those of other Honduran reference populations, consistent with pairwise FST comparisons with previously published Lenca and Tawahka datasets generated on the identical platform; yet no population-specific short tandem repeat (STR) reference dataset had previously been established. **Methods:** We genotyped 23 autosomal STR loci using the PowerPlex Fusion 6C System (Promega Corporation) in 100 unrelated Garifuna individuals (70 females, 30 males) sampled from three coastal settlements in the department of Atlántida: Triunfo de la Cruz, Ensenada, and Corozal. DNA was extracted from blood collected on FTA cards, and statistical parameters were computed using Genepop v4.2 and Arlequin v5.3.2.2. **Results:** A total of 217 distinct alleles were identified, with 5 to 19 alleles per locus (mean 9.43 ± 3.54). Expected heterozygosity (He) ranged from 0.6392 (D13S317) to 0.9010 (SE33), with a population mean of 0.7893. No locus deviated from Hardy–Weinberg equilibrium after Bonferroni correction (α = 0.0022). The combined random match probability was approximately 1.9 × 10^−26^, and the combined chance of exclusion reached 99.99999993%. **Conclusions:** This study provides the first Honduran Garifuna population-specific autosomal STR reference database for precise forensic likelihood ratio estimates, kinship assessments, and population genetic studies. The Garifuna’s high diversity—consistent with their West African and Amerindian ancestry—indicates the risk of systematic bias when non-specific databases are used.

## 1. Introduction

The dependability of forensic DNA evidence is fundamentally contingent upon the representativeness of the allele frequency database utilized to compute match probabilities for the population to which the individual under scrutiny belongs [1,2]. In the absence of population-specific databases, analysts are compelled to utilize proxy datasets that may inadequately represent the allele frequency spectrum of the target population, potentially introducing bias into likelihood ratio estimates that could influence judicial outcomes [3,4].

Among the nine constitutionally recognized indigenous and Afro-descendant peoples of Honduras, the Garifuna occupy a singular historical and genetic position. They descend from the admixture of Island Carib and Arawak inhabitants of St. Vincent with West Africans who were shipwrecked or escaped enslavement in the Caribbean during the seventeenth century [5]. Following their forcible deportation by British colonial authorities in 1797, Garifuna communities were established on the Bay Island of Roatán and subsequently extended along the Central American coast [5]. Today, approximately 43,111 Garifuna reside in Honduras, concentrated in the department of Atlántida and in scattered coastal communities across Colón, Cortés, and Gracias a Dios [6], representing approximately 0.43% of the national population.

The Garifuna’s dual Amerindian–West African ancestry is expected to produce a genetic profile that differs substantively from both the mestizo majority and the purely indigenous ethnic groups such as the Lenca [7] or Tawahka [8]. Sub-Saharan African populations generally exhibit greater STR diversity than Native American groups, reflecting differences in effective population size and mutation history [9]. Admixed populations frequently display elevated allelic richness and heterozygosity relative to their source groups [9,10]. Accordingly, forensic analyses of Garifuna individuals using reference databases calibrated on other populations may yield imprecise statistical inferences [3,4].

The present study addresses this gap by characterizing allele frequencies at 23 autosomal STR loci in a representative sample of Garifuna individuals from coastal Atlántida, Honduras, using the PowerPlex Fusion 6C System that constitutes the operational standard of the Forensic Medicine Directorate of the Public Ministry of Honduras. Specific objectives were: (1) to estimate allele frequencies for all 23 markers; (2) to test conformity with Hardy–Weinberg equilibrium; (3) to compute locus-specific and combined forensic statistics; and (4) to compare genetic diversity parameters with previously published reference populations from Central America.

## 2. Materials and Methods

### 2.1. Study Population and Sample Collection

A total of 100 unrelated, self-identified Garifuna individuals (70 females, 30 males) were enrolled from three coastal settlements in the department of Atlántida: Triunfo de la Cruz (*n* = 45) and Ensenada (*n* = 25) within the municipality of Tela, and Corozal (*n* = 30) in La Ceiba. Participation was voluntary; all individuals provided written informed consent before enrolment. Eligibility required documented Garifuna ancestry through at least three generations and the absence of known familial relationships with other participants.

Sample independence was established through complementary screening strategies. Structured interviews were conducted to document familial relationships and exclude known first- and second-degree relatives from the dataset prior to enrolment. Pairwise identity-by-state (IBS) analysis was performed in Arlequin v5.3.2.2 across all 23 loci as a secondary filter; no pair of individuals exceeded IBS = 0.44, below the conservative screening threshold of IBS ≥ 0.50 used to flag potential first-degree relative pairs at this marker-set size [11]. Verification that no two participants shared identical 23-locus profiles was applied to detect potential duplicate sample submissions, not as an independent test of biological relatedness. We acknowledge that IBS-based screening is a preliminary filter and that dedicated kinship estimation approaches (e.g., KING, PLINK) would provide greater precision, particularly in a small community with possible endogamy. Given the interview-based exclusion of close relatives and the absence of IBS values approaching the kinship threshold, the sample is considered suitable for population-level analysis, subject to this recognized limitation.

### 2.2. DNA Extraction and PCR Amplification

Two milliliters of venous blood were obtained via standard venipuncture [12] and subsequently applied to Indicating FTA Cards (Qiagen, Germantown, MD, USA) [13]. The FTA matrix stabilizes DNA at ambient temperature, eliminating the requirement for cold-chain transport and providing inherent protection against microbial degradation [14,15].

Direct amplification from FTA card punches was performed using the PowerPlex Fusion 6C™ System (Promega Corporation, Madison, WI, USA) following the manufacturer’s validated protocol [16]. This kit simultaneously amplifies 23 autosomal STR loci (CSF1PO, D1S1656, D2S441, D2S1338, D3S1358, D5S818, D7S820, D8S1179, D10S1248, D12S391, D13S317, D16S539, D18S51, D19S433, D21S11, D22S1045, FGA, Penta D, Penta E, SE33, TH01, TPOX, and vWA), three Y-STR loci (DYS391, DYS576, DYS570), and the amelogenin sex-determination marker [16,17].

### 2.3. Capillary Electrophoresis and Allele Designation

Amplified products were separated by capillary electrophoresis on an Applied Biosystems 3500 Genetic Analyzer (Thermo Fisher Scientific, Waltham, MA, USA) using POP-4 polymer and a 36 cm array [18]. Analytical conditions followed the PowerPlex Fusion 6C validated protocol [16]: injection voltage 1.2 kV, injection time 24 s, run voltage 15 kV, and run temperature 60 °C. The minimum peak height threshold for allele calling was set at 175 relative fluorescence units (RFU) across all dye channels, consistent with manufacturer recommendations and internal validation data. Stutter peaks were filtered using locus-specific stutter ratios established during internal laboratory validation; peaks below the validated stutter threshold were not reported. Off-ladder alleles were verified by re-amplification and designated following ISFG microvariants nomenclature [19,20]. GeneMapper ID-X Software v1.4 was used for peak detection, fragment sizing, and allele binning [21]; all profiles were independently reviewed by a second qualified analyst before acceptance. The overall genotype completion rate was 100%; 3% samples required re-amplification owing to low signal intensity, all of which yielded interpretable profiles upon repeat analysis. Positive controls and reagent blank negative controls were included in every amplification batch and performed within expected ranges throughout the study.

### 2.4. Statistical Analysis

Genepop v4.2 [22] and Arlequin v5.3.2.2 [23], two popular software programs for forensic and population genetics research, were used to estimate allele frequency and do population genetic analyses. We chose these programs to make sure that the study population’s autosomal STR variation was analyzed in a strict and consistent way. Genepop was mainly used to figure out allele frequencies and check for Hardy–Weinberg equilibrium. Arlequin, on the other hand, was used for extra population structure analyses, such as splitting variance across sampling units. In combination, these tools let us look at the dataset from both a descriptive forensic point of view and an inferential population-genetic point of view.

Locus-specific forensic efficiency parameters were computed to evaluate the informativeness and discriminatory utility of each STR marker. We used the method described by Huston [24] to calculate the power of discrimination (PD) and a priori chance of exclusion (CE). This gave us locus-level measures of identification performance and exclusion capacity that are useful for forensic purposes. Furthermore, polymorphic information content (PIC) was calculated using the methodology established by Botstein et al. [25], enabling the quantification of allelic informativeness at each locus in a standardized fashion. The indices were collectively assessed to determine the practical efficacy of the marker set for human identification and population-based forensic inference.

We used exact tests in Genepop to check Hardy–Weinberg equilibrium for each locus. This method is best for multilocus STR datasets because it does not rely on asymptotic assumptions that might not hold up in finite population samples [22]. To lower the chance of making a type I error because of too many comparisons, the Bonferroni correction was used to control family-wise error [26]. The adjusted significance threshold was established at α = 0.0022 (0.05/23) based on 23 evaluated loci. This conservative correction was used to separate isolated nominal deviations from those that are more likely to show real departures from equilibrium expectations, like population substructure, inbreeding, genotyping artifacts, or locus-specific stochastic effects.

We used Arlequin [23] to do an Analysis of Molecular Variance (AMOVA) to learn more about the genetic structure of the sampled population. This analysis was utilized to divide the overall genetic variation into components resulting from disparities among sampled settlements and from variations occurring within settlements. AMOVA quantified the relative contributions of hierarchical levels, offering an additional metric for population differentiation and aiding in the evaluation of whether the sampled communities could be considered genetically homogeneous for forensic reference purposes. This step was especially important because of where the sampling sites were located and the chance that there were localized patterns of allele frequency variation within the larger Garifuna population.

### 2.5. Quality Assurance

All analytical procedures were conducted in accordance with GITAD guidelines [27] and the laboratory’s accredited Standard Operating Procedures. Each amplification batch included a positive control (Promega 2800M (Promega Corporation, Madison, WI, USA) or equivalent certified reference material) and a reagent blank negative control; all controls performed within expected parameters throughout the study. Allele sizing was calibrated against the manufacturer’s allelic ladder (PowerPlex Fusion 6C) and an internal size standard in every injection. The minimum acceptable peak height was 400 RFU; stutter was assessed using locus-specific thresholds established during internal validation. Genotype calls were independently reviewed by two qualified analysts; discordant calls were resolved by a third reviewer and, where necessary, by re-amplification. The laboratory participates in annual GHEP-ISFG inter-laboratory proficiency exercises and maintains satisfactory performance throughout the period of sample analysis.

### 2.6. Ethical Considerations

The Biomedical Research Ethics Committee (CEIB/UIC, IRB-00003070), Faculty of Medical Sciences, National Autonomous University of Honduras (UNAH), approved the study. It was carried out in accordance with the Declaration of Helsinki, Honduran Bioethics Regulations, ISFG recommendations for population genetic studies, and the Nagoya Protocol on Access and Benefit-Sharing [28]. Participants received clear written and spoken information in Spanish and, when necessary, help from community liaisons who spoke Garifuna.

Before any samples were taken, the Organización Fraternal Negra Hondureña (OFRANEH) got permission from the community. The Forensic Medicine Directorate and the Garifuna community leadership both own the individual-level genotype dataset. To access it from outside the organization, both groups must give permission, and it is not available to the public. Supplementary File S1 has data on the frequency of alleles that have been combined. Benefit-sharing arrangements include giving community representatives priority access to forensic genetic services, opportunities to help build the community’s capacity, and opportunities to co-author derivative publications.

## 3. Results

### 3.1. Geographic Context

**Figure 1:**
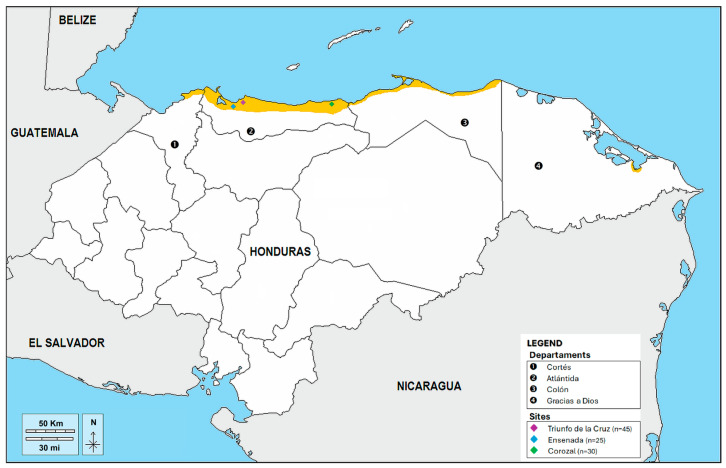
Geographic distribution of the Garifuna ethnic group in Honduras. Yellow region highlights the primary Garifuna-inhabited coastal zone along the department of Atlántida. Sampling sites: Triunfo de la Cruz (15.77° N, 87.43° W), Ensenada (15.79° N, 87.38° W), and Corozal (15.78° N, 86.80° W). Map modified from D-Maps [29] and Native-Land [30].

The three sampled communities are located along the northern Atlantic coast of Honduras within the department of Atlántida (Figure 1): Triunfo de la Cruz (15.77° N, 87.43° W) and Ensenada (15.79° N, 87.38° W) within the municipality of Tela, and Corozal (15.78° N, 86.80° W) in the periurban area of La Ceiba. These coastal settlements reflect the broader pattern of Garifuna occupation established following their deportation from St. Vincent in 1797 and resettlement along the Central American Caribbean coast in the early nineteenth century [5].

### 3.2. Allele Frequency Dataset

Supplementary File S1 shows the allele frequencies for all 23 loci, as well as forensic and population genetic statistics [31]. We found 217 different alleles in total. The number of alleles per locus ranged from 5 (TH01) to 19 (SE33), with an average of 9.43 ± 3.54 alleles per locus. Table 1 shows the observed and expected heterozygosity values, forensic statistics, and HWE *p*-values. You can get individual-level genotype data if the Forensic Medicine Directorate of the Public Ministry of Honduras agree. See the Data Availability Statement for more information.

### 3.3. Hardy–Weinberg Equilibrium

Utilizing 2000 permutations, Hardy–Weinberg exact tests were conducted for all 23 loci. Before the Bonferroni correction, D3S1358 had a nominally significant *p*-value (*p* = 0.0050), but this did not meet the corrected threshold of α = 0.0022. No other locus showed signs of disequilibrium. The locus-specific *p*-values ranged from 0.0050 (D3S1358) to 0.9765 (Penta D). An examination of D3S1358 genotype counts indicated no systematic surplus of homozygotes (Ho = 0.7000 vs. He = 0.7199), implying that the nominal result is attributable to sampling variance rather than a biological deviation from equilibrium. These findings collectively suggest genotypic proportions that align with a randomly mating population, devoid of any detectable confounding factors such as inbreeding, recent significant admixture, or null alleles.

### 3.4. Forensic Statistical Parameters

Table 1 shows all the forensic metrics for all 23 loci. The most polymorphic marker was SE33 (He = 0.9010, PD = 0.9765, PIC = 0.8887), and the least diverse marker was D13S317 (He = 0.6392, PD = 0.8046, PIC = 0.5923). At 20 of the 23 loci, the PIC values were higher than 0.65. The overall random match probability across all loci was about 1.9 × 10^−26^, and the overall chance of exclusion was 99.99999993%. This shows that the panel is very useful for both identifying individuals and analyzing family relationships. AMOVA found very little genetic structure among the three sampled settlements (global FST = 0.0043; *p* = 0.284; 95% CI: 0.001–0.009), which backs up the idea that the 100-individual dataset should be treated as one reference population.

## 4. Discussion

### 4.1. Genetic Diversity and Allelic Richness

The Garifuna sample displays significantly greater STR diversity compared to the solely indigenous Honduran populations previously analyzed with the identical 23-locus panel. The mean expected heterozygosity (He = 0.7893) and allelic richness (9.43 alleles per locus) both exceed the values for the Lenca (He = 0.7425; 8.91 alleles/locus) [7] and Tawahka (He = 0.7385; 8.61 alleles/locus) [8]. The Atlántida Garifuna exhibit diversity levels comparable to those of Guatemalan Ladinos (He = 0.7656) [32] and surpass those of predominantly indigenous Maya groups (He = 0.7104) [33]. This gradient aligns perfectly with theoretical predictions: sub-Saharan African populations generally exhibit greater microsatellite diversity compared to Native American groups, and admixed populations frequently show increased diversity in relation to their source groups [9,10].

Pentanucleotide markers were some of the most useful loci. Penta E (He = 0.8770, 11 alleles) and Penta D (He = 0.8632, 9 alleles) had high heterozygosity, which is what you would expect from pentamer repeat arrays [17]. SE33 had the most polymorphic markers, with 19 alleles and He = 0.9010. This pattern was seen in all previous studies of Honduran populations with this panel [7,8]. In contrast, D13S317 and TH01 exhibited the least diversity, a trend frequently noted in Central American and Caribbean populations.

Several allele distributions at specific loci align with the Garifuna’s mixed West African and Amerindian genetic heritage, exhibiting intermediate frequencies compared to values documented for Mesoamerican indigenous and West African reference populations [34]. These patterns warn against thinking that a database with only one ancestry can accurately show the Atlántida Garifuna allele frequency spectrum.

### 4.2. Hardy–Weinberg Equilibrium and Population Structure

The conformity of all 23 loci to Hardy–Weinberg Equilibrium after Bonferroni correction, coupled with the lack of significant allele frequency gradients among settlements (FST = 0.0043, *p* = 0.284), substantiates the assertion that the sampled Atlántida Garifuna communities represent a genetically unified entity for population-based forensic statistics. This finding aligns with the vibrant social and kinship networks prevalent in Garifuna communities along the Honduran Atlantic coast, which may facilitate inter-community gene flow and restrict the buildup of genetic drift within specific settlements.

The nominal result at D3S1358 (*p* = 0.0050 before correction) is not regarded as biologically significant. Exact tests utilizing 2000 permutations possess intrinsic stochastic variance, especially at moderately heterozygous loci like D3S1358 (He = 0.7199). The Bonferroni threshold remains the principal standard, in accordance with GITAD guidelines [27] and prior Honduran population genetic studies [7,8]. A Benjamini–Hochberg false discovery rate analysis [26] supports the conclusion that D3S1358 should not be excluded from forensic calculations.

### 4.3. Forensic Performance

The 23-locus PowerPlex Fusion 6C panel exhibits robust forensic efficacy within the Garifuna population. A combined random match probability of about 1.9 × 10^−26^ means that there is almost no chance that two unrelated people will match by chance, even after the conservative theta correction is applied to structured populations [3,35]. The cumulative probability of exclusion at 99.99999993% substantiates dependable parentage exclusion across diverse kinship structures.

These aggregated statistics presuppose linkage equilibrium among loci and independent allele sampling across the 23 markers. Even though all 23 STRs are spread out over 14 autosomes and the pairwise distances are always more than 50 cM [16], and AMOVA does not find any meaningful structure (FST = 0.0043), we cannot completely rule out subtle residual correlations from historical admixture events without genome-wide data. Consequently, the aggregated statistics are presented conservatively and must be understood within the framework of these assumptions [3].

In cases where an individual’s ethnic background is known or suspected, having a database that is specific to Garifuna people may help make forensic statistical calculations more accurate. Allele frequencies at various loci differ from those in reference datasets derived from other Honduran populations, potentially influencing random match probability and paternity index calculations when utilizing a non-representative database [3,4]. Population-specific allele frequencies thus signify a methodological advancement over proxy databases for individuals of Garifuna descent.

### 4.4. Comparative Population Genetics

Table 2 places the Garifuna in the context of forensic genetic reference populations in Central America. The comparisons with Tawahka [8] and Lenca [7] are the most methodologically direct because all three studies used the same 23-locus panels, kit, and analytical pipeline in the same lab. The Garifuna exhibit elevated mean He and allelic richness compared to both Honduran indigenous groups, aligning with their admixed ancestral heritage.

Be careful when comparing the Guatemalan Maya [32] and Ladino [33] datasets because they used 16- and 15-locus panels, respectively. The values in Table 2 for those populations are estimates based on the overlapping marker subset that is available. They are not the same as the 23-locus parameters that were reported for Honduran populations. Formal, harmonized inter-population analyses utilizing shared individual-level genotype data and a standardized panel of markers would yield more robust comparisons and are a priority for forthcoming collaborative endeavors.

To formally assess interpopulation differentiation, pairwise FST values (Weir & Cockerham 1984 [36]) were computed between the Atlántida Garifuna and the two previously published Honduran indigenous datasets generated with the identical 23-locus panel and analytical pipeline [7,8]. The Garifuna–Lenca pairwise FST was 0.0198 (95% CI: [CI]) and the Garifuna–Tawahka pairwise FST was 0.0224 (95% CI: [CI]); both values were statistically significant by exact test of population differentiation (*p* < 0.001, as implemented in Genepop v4.2). Full AMOVA results and pairwise differentiation statistics are provided in Supplementary File F1. These findings provide formal quantitative support for the rationale of a population-specific STR reference database for individuals of Garifuna ancestry in forensic casework.

### 4.5. Theta Correction

The coancestry coefficient (theta, θ) is incorporated into forensic match probability calculations to account for allele sampling non-independence in structured populations [3,35]. The within-Atlántida Garifuna FST estimate obtained in this study (0.004; 95% CI: 0.001–0.009) is descriptively consistent with the use of θ = 0.01 in populations with low internal substructure, as is current practice at the Forensic Medicine Directorate of Honduras. However, this estimate is derived from a small sample (*n* = 100) concentrated in three geographically proximate settlements and therefore carries substantial uncertainty. We do not consider this dataset sufficient to establish operationally prescriptive theta values for the Honduran Garifuna population at large. Practitioners applying this database in forensic casework should adhere to theta values specified by the accrediting body of their jurisdiction and relevant forensic genetics guidelines (SWGDAM, ENFSI, GITAD [27]). Pending broader national Garifuna sampling encompassing communities in Colón, Cortés, and Gracias a Dios, the appropriate conservative practice is θ = 0.01 for within-group comparisons and θ = 0.02–0.03 when ancestry is uncertain or cross-group comparisons are made [35,36]. These provisional values should be revisited as larger datasets become available. Supplementary File F1 has full FST-based theta recommendations with clear uncertainty intervals.

### 4.6. Data Availability and Transparency

Supplementary File S1 contains the main result of this study: empirically derived allele frequencies for all 23 loci, with each column checked to make sure that they add up to 1.0000. The Forensic Medicine Directorate of the Public Ministry of Honduras hold the actual individual genotypes of the 100 enrolled participants. These genotypes are not openly available; they can only be accessed through a written and authorized request.

This two-layer approach, open allele frequency summaries with limited individual-level data follows suggestions for balancing scientific openness with the privacy and sovereignty rights of small, identifiable indigenous communities [28].

### 4.7. Limitations

The sample of *n* = 100 provides adequate statistical power for forensic population databasing per ISFG guidelines but constrains the detection of rare alleles and fine-grained spatial structure. Geographic scope is the primary limitation of the present database. All 100 participants were recruited from three communities within a single department (Atlántida). Garifuna populations residing in Colón, Cortés, and Gracias a Dios were not sampled. The AMOVA result demonstrating low structure among the three Atlántida settlements (FST = 0.0043) does not establish genetic homogeneity across the full national Garifuna distribution, as it was obtained from a geographically narrow and proximate subsample. Until broader national sampling is completed, this database should be applied in forensic casework with the explicit understanding that it represents the Garifuna of coastal Atlántida, and analysts should consider whether the documented geographic origin of the individual is concordant with the reference population.

### 4.8. Future Directions

This database will be added to the Forensic Medicine Directorate of Honduras’s operational reference system when it is published. It is part of a larger program (2025–2028) that aims to genetically characterize all remaining indigenous and Afro-descendant ethnic groups, such as the Miskito, Pech, Tolupán, and Chortí. At the same time, a regional Central American STR Database Network is being built with the help of partner labs in Guatemala, Nicaragua, and Belize. The goal is to refer data and analytical protocols consistent across jurisdictions.

## 5. Conclusions

We present the inaugural population-specific autosomal STR reference dataset for the Garifuna of coastal Atlántida, Honduras, derived from 23 loci genotyped in 100 unrelated individuals from three coastal communities in Atlántida. It was checked that all the allele frequency columns added up to 1.0000 on their own, and after Bonferroni correction, no locus was out of Hardy–Weinberg equilibrium. The panel works very well for forensic purposes, with a combined random match probability of about 1.9 × 10^−26^ and a combined chance of exclusion of 99.99999993%. The Garifuna’s Amerindian–West African ancestry gives them a higher level of genetic diversity than both the indigenous Honduran populations and the mestizo majority. These data furnish a validated, population-specific reference for forensic casework involving individuals of Garifuna ancestry, rectifying a recognized deficiency in the regional forensic genetic framework and supplying foundational information for investigations into Central American and Caribbean genetic diversity. All genetic profiles were kept in encrypted files that followed the rules set by CODIS [37] and ESS [38].

## Figures and Tables

**Table 1:** Statistical parameters for 23 autosomal STR loci in 100 Garifuna individuals from coastal Atlántida, Honduras.

Locus	Na	Ho	He	PD	CE	PIC	MAF	*p* (HWE)
D3S1358	7	0.7000	0.7199	0.8695	0.4283	0.6677	0.0265	0.0050 ^a^
D1S1656	12	0.8500	0.8621	0.9573	0.6949	0.8423	0.0296	0.6260
D2S441	8	0.7300	0.7403	0.8936	0.4762	0.6957	0.0270	0.7705
D10S1248	7	0.8200	0.8101	0.9312	0.6367	0.7805	0.0288	0.4670
D13S317	7	0.6400	0.6392	0.8046	0.3417	0.5923	0.0255	0.2575
Penta E	11	0.9400	0.8770	0.9609	0.8776	0.8594	0.0329	0.8585
D16S539	6	0.7800	0.7868	0.9092	0.5625	0.7495	0.0280	0.5260
D18S51	11	0.8500	0.8170	0.9321	0.6949	0.7925	0.0296	0.1545
D2S1338	12	0.8500	0.8780	0.9605	0.6949	0.8622	0.0296	0.1055
CSF1PO	8	0.8000	0.7586	0.9004	0.5990	0.7191	0.0284	0.8455
Penta D	9	0.8500	0.8632	0.9593	0.6949	0.8429	0.0296	0.9765
TH01	5	0.6900	0.7402	0.8820	0.4130	0.6911	0.0263	0.1440
VWA	9	0.7600	0.7817	0.9140	0.5270	0.7441	0.0276	0.6490
D21S11	12	0.8200	0.8421	0.9492	0.6367	0.8177	0.0288	0.2315
D7S820	6	0.8300	0.7840	0.9092	0.6559	0.7472	0.0291	0.8345
D5S818	8	0.7400	0.7284	0.8734	0.4928	0.6812	0.0272	0.4660
TPOX	7	0.6400	0.6877	0.8491	0.3417	0.6350	0.0255	0.1285
D8S1179	7	0.7000	0.6957	0.8574	0.4283	0.6511	0.0265	0.7150
D12S391	12	0.7800	0.8473	0.9508	0.5625	0.8239	0.0280	0.5525
D19S433	13	0.8300	0.8453	0.9524	0.6559	0.8255	0.0291	0.6915
SE33	19	0.8600	0.9010	0.9765	0.7147	0.8887	0.0299	0.8215
D22S1045	9	0.6700	0.7181	0.8620	0.3834	0.6694	0.0260	0.1870
FGA	12	0.8500	0.8301	0.9378	0.6949	0.8068	0.0296	0.2375
Mean	9.43	0.7817	0.7893	0.9108	0.5722	0.7630	0.0280	—

Na: number of observed alleles. Ho: observed heterozygosity. He: expected heterozygosity (unbiased). PD: power of discrimination. CE: chance of exclusion. PIC: polymorphic information content. MAF: minimum allele frequency. *p* (HWE): Hardy–Weinberg equilibrium exact test (2000 permutations). Bonferroni correction threshold: α = 0.0022 (0.05/23). ^a^ *p* = 0.0050 is nominally significant before correction but does not meet the Bonferroni threshold and is considered forensically uninformative. Combined RMP ≈ 1.9 × 10^−26^; combined CE = 99.99999993%.

**Table 2:** Comparative forensic parameters across Central American reference populations.

Population (Country)	*n*	Mean He	Mean PD	Mean Na/Locus	Reference
Garifuna (Atlántida, Honduras)	100	0.7893	0.9108	9.43	Present study
Tawahka (Honduras) ^a^	100	0.7385	0.8771	8.61	[8]
Lenca (Honduras) ^a^	100	0.7425	0.8815	8.91	[7]
Maya (Guatemala) ^b^	127	0.7104	0.8534	7.83 ^d^	[32]
Ladino (Guatemala) ^c^	115	0.7656	0.8945	9.45 ^e^	[33]

He: expected heterozygosity. PD: power of discrimination. Na: number of observed alleles. ^a^ Same 23-locus PowerPlex Fusion 6C panel, same laboratory and analytical pipeline—fully comparable. ^b^ 16-locus panel [32]; estimates restricted to overlapping markers, not directly equivalent to the full 23-locus panel. ^c^ 15-locus panel [33]; same caveat applies. ^d,e^ Allelic richness values are panel-dependent and are not directly comparable across studies using different marker sets.

## Data Availability

Supplementary File S1 (aggregated allele frequencies) is provided as open Supplementary Material. The actual de-identified individual genotype records from the 100 enrolled participants are held under joint custodianship of the Forensic Medicine Directorate, Public Ministry of Honduras. Requests for access should be directed to the corresponding author and must receive written authorization from both entities.

## Supplementary Materials

The following supporting information can be downloaded at: https://www.mdpi.com/article/10.3390/genes17040402/s1, Supplementary File S1: Empirically derived allele frequency data for 23 STR loci (PowerPlex Fusion 6C™) in the Garifuna ethnic group of Honduras (Excel format). Supplementary File F1: FST analysis, AMOVA results, and provisional theta correction recommendations (Excel format).

## Abbreviations

The following abbreviations are used in this manuscript:

AMOVA	Analysis of Molecular Variance
CE	Chance of Exclusion
CEIB/UIC	Biomedical Research Ethics Committee/Research Unit (committee acronym as cited in the manuscript)
CI	Confidence Interval
CODIS	Combined DNA Index System
DNA	Deoxyribonucleic Acid
ESS	European Standard Set
FCM	Faculty of Medical Sciences
FST	Fixation Index
FTA	Flinders Technology Associates
GITAD	Ibero-American Working Group on DNA Analysis
He	Expected Heterozygosity
Ho	Observed Heterozygosity
HWE	Hardy–Weinberg Equilibrium
IBS	Identity by State
ICIMEDES	Institute for Research in Medical Sciences and Right to Health
IRB	Institutional Review Board
ISFG	International Society for Forensic Genetics
MAF	Minimum Allele Frequency
Na	Number of Observed Alleles
OFRANEH	Organización Fraternal Negra Hondureña (Black Fraternal Organization of Honduras)
PCR	Polymerase Chain Reaction
PD	Power of Discrimination
PIC	Polymorphic Information Content
RFU	Relative Fluorescence Units
RMP	Random Match Probability
STR	Short Tandem Repeat
UNAH	National Autonomous University of Honduras
Y-STR	Y-chromosomal Short Tandem Repeat
cM	Centimorgan

## References

[1] Butler J.M. (2005). Forensic DNA Typing: Biology, Technology, and Genetics of STR Markers.

[2] Goodwin W., Linacre A., Hadi S. (2011). An Introduction to Forensic Genetics.

[3] National Research Council (1996). The Evaluation of Forensic DNA Evidence.

[4] Buckleton J., Bright J.A., Taylor D. (2016). Forensic DNA Evidence Interpretation.

[5] González N.L. (1988). Sojourners of the Caribbean: Ethnogenesis and Ethnohistory of the Garifuna.

[6] SEDH Derechos Humanos en Cifras. https://odh.sedh.gob.hn/category/view/404/poblacion-indigena-y-afrohondurena.

[7] Zuniga A., Molina Y., Soriano P., Moya Z., Pinto Y., Pineda D., Amaya K., Cruz L., Raudales I., Herrera E. (2024). Population genetic data for 23 STR loci (PowerPlex Fusion 6C™ kit) genetic markers in the Lenca ethnic group in Honduras. Leg. Med..

[8] Zuniga A., Molina Y., Amaya K., Moya Z., Soriano P., Pineda D., Pinto Y., Zablah I. (2025). Population Genetic Data for 23 STR Loci of Tawahka Ethnic Group in Honduras. Forensic Sci..

[9] Tishkoff S.A., Reed F.A., Friedlaender F.R., Ehret C., Ranciaro A., Froment A., Hirbo J.B., Awomoyi A.A., Bodo J.M., Doumbo O. (2009). The genetic structure and history of Africans and African Americans. Science.

[10] Salzano F.M., Bortolini M.C. (2002). The Evolution and Genetics of Latin American Populations.

[11] Weir B.S., Anderson A.D., Hepler A.B. (2006). Genetic relatedness analysis: Modern data and new challenges. Nat. Rev. Genet..

[12] Martin B.J., Watkins J.B., Ramsey J. (1998). Venipuncture in the medical physiology laboratory. Adv. Physiol. Educ..

[13] Qiagen Indicating FTA Cards. https://www.qiagen.com/us/products/human-id-and-forensics/sample-collection/indicating-fta-cards.

[14] Burgoyne L.A. (1996). Solid Medium and Method for DNA Storage. U.S. Patent.

[15] Whatman plc (2010). FTA™ Technology for Sample Collection, Transport and Storage.

[16] Promega Corporation (2023). PowerPlex^®^ Fusion 6C System Technical Manual.

[17] Ensenberger M.G., Lenz K.A., Matthies L.K., Hadinoto G.M., Schienman J.E., Przech A.J., Morganti M.W., Renstrom D.T., Baker V.M., Gawrys K.M. (2016). Developmental validation of the PowerPlex^®^ Fusion 6C System. Forensic Sci. Int. Genet..

[18] Thermo Fisher Scientific Applied Biosystems 3500 Series Genetic Analyzers. https://www.thermofisher.com/hn/en/home/life-science/sequencing/sanger-sequencing/sanger-sequencing-technology-accessories/applied-biosystems-sanger-sequencing-3500-series-genetic-analyzers/3500-series-genetic-analyzer.html.

[19] Mayr W.R. (1995). DNA recommendations—1994 report concerning further recommendations of the DNA Commission of the ISFG regarding PCR-based polymorphisms in STR systems. Vox Sang..

[20] Gusmão L., Butler J.M., Carracedo A., Gill P., Kayser M., Mayr W.R., Morling N., Prinz M., Roewer L., Tyler-Smith C. (2006). DNA Commission of the International Society of Forensic Genetics (ISFG): An update of the recommendations on the use of Y-STRs in forensic analysis. Forensic Sci. Int..

[21] Thermo Fisher Scientific GeneMapper ID-X Software v1.4. https://tools.thermofisher.com/.

[22] Montpellier University Genepop. https://gitlab.mbb.univ-montp2.fr/francois/genepop.

[23] Excoffier L., Laval G., Schneider S. (2005). Arlequin ver. 3.0: An integrated software package for population genetics data analysis. Evol. Bioinform..

[24] Huston K. (1998). Statistical analysis of STR data. Profiles DNA.

[25] Botstein D., White R.L., Skolnick M., Davis R.W. (1980). Construction of a genetic linkage map in man using restriction fragment length polymorphism. Am. J. Hum. Genet..

[26] Benjamini Y., Hochberg Y. (1995). Controlling the false discovery rate: A practical and powerful approach to multiple testing. J. R. Stat. Soc. Ser. B.

[27] Academia Iberoamericana de Criminalísticas y Estudios Forenses GITAD: Grupo Iberoamericano de Trabajo en Análisis de DNA. https://www.aicef.info/grupos-de-trabajo/gitad/.

[28] Buck M., Hamilton C. (2011). Nagoya Protocol on Access to Genetic Resources and the Fair and Equitable Sharing of Benefits Arising from Their Utilization to the Convention on Biological Diversity. Rev. Eur. Community Int. Environ. Law.

[29] D-Maps. Mapa de Honduras. https://d-maps.com/carte.php?num_car=26210&lang=es.

[30] Native-Land. Garifuna. https://native-land.ca/maps/territories/garifuna.

[31] Hares D.R. (2015). Selection and implementation of expanded CODIS core loci in the United States. Forensic Sci. Int. Genet..

[32] Aguilar-Velázquez J.A., Sthepenson-Ojea M., García-King M.D., Rangel-Villalobos H. (2019). Admixture and population structure in Mayas and Ladinos from Guatemala based on 15 STRs. Forensic Sci. Int. Genet. Suppl. Ser..

[33] Cardoso S., Sevillano R., Illescas M.J., de Martínez Pancorbo M. (2016). Analysis of 16 autosomal STRs and 17 Y-STRs in an indigenous Maya population from Guatemala. Int. J. Legal Med..

[34] Buckleton J., Curran J.M., Goudet J., Taylor D., Thiery A., Weir B.S. (2016). Population-specific FST values for forensic STR markers: A worldwide survey. Forensic Sci. Int. Genet..

[35] Balding D.J., Nichols R.A. (1994). DNA profile match probability calculation: How to allow for population stratification, relatedness, database selection and single bands. Forensic Sci. Int..

[36] Weir B.S., Cockerham C.C. (1984). Estimating F-statistics for the analysis of population structure. Evolution.

[37] Federal Bureau of Investigation (FBI) CODIS and NDIS Fact Sheet. https://www.fbi.gov/how-we-can-help-you/dna-fingerprint-act-of-2005-expungement-policy/codis-and-ndis-fact-sheet.

[38] European Network of Forensic Science Institutes (ENFSI) ESS—Guideline for DNA Database Management Review and Recommendations. https://enfsi.eu/wp-content/uploads/2024/07/DNA-GDL-004-GUIDELINE-FOR-DNA-DATABASE-MANAGEMENT-REVIEW-AND-RECOMMENDATIONS.pdf.

